# The Bergen 4-day treatment for panic disorder: adapting to COVID-19 restrictions with a hybrid approach of face-to-face and videoconference modalities

**DOI:** 10.1186/s12888-023-05062-7

**Published:** 2023-08-08

**Authors:** Kay Morten Hjelle, Thorstein Olsen Eide, Anders Lillevik Thorsen, Gerd Kvale, Kristen Hagen, Ivar Snorrason, Thröstur Björgvinsson, Bjarne Hansen

**Affiliations:** 1 Bergen Center for Brain Plasticity, Haukeland University Hospital, Bergen, Norway; 2 Center for Crisis Psychology, Faculty of Psychology, University of Bergen, Bergen, Norway; 3 Møre and Romsdal Hospital Trust, Molde, Norway; 4 Department of Clinical Psychology, University of Bergen, Bergen, Norway; 5 Center for OCD and Related Disorders, Massachusetts General Hospital, Boston, USA; 6 Harvard Medical School, Boston, USA; 7 McLean Hospital, Belmont, USA

**Keywords:** Panic disorder, Concentrated treatment, Exposure, B4DT, CBT

## Abstract

**Background:**

The Bergen 4-day treatment (B4DT) is a concentrated exposure-based therapy that has been shown to be effective in the treatment of anxiety disorders. The current study sought to examine the effectiveness of B4DT for panic disorder (PD), when delivered with a combination of face-to-face sessions and videoconferencing.

**Methods:**

Treatment was delivered to 50 patients from April 2020 to May 2021. Because of regulations during the pandemic, a significant portion of the treatment was conducted via videoconference. The primary outcome measure was the clinician-rated Panic Disorder Severity Scale (PDSS), and secondary measures included patient-rated symptoms of panic disorder, agoraphobia, generalized anxiety, depression, and treatment satisfaction. Changes in symptom levels over time were estimated using multilevel models.

**Results:**

Patients showed a significant reduction in clinician-rated symptoms of panic disorder (Measured by PDSS) from before treatment to post treatment (d = 2.18) and 3-month follow-up (d = 2.01). At three months follow-up 62% of patients were classified as in remission, while 70% reported a clinically significant response. We also found a reduction in symptoms of depression and generalized anxiety, and the patients reported high satisfaction with the treatment.

**Conclusion:**

The current study suggests that B4DT delivered in a combination of videoconference and face-to-face meetings may be a useful treatment approach. As the study is uncontrolled, future studies should also include more strictly designed investigations.

## Introduction

Panic Disorder (PD) is characterized by the occurrence of sudden and intense panic attacks. Panic attacks are followed by a fear of physical symptoms and fear of catastrophic consequences of future panic attacks. Patients therefore engage in excessive safety behaviours, avoidance and worry of new panic attacks, which can result in a vicious circle which often impairs functioning and reduces quality of life [1, 2]. Agoraphobia, the fear of being in situations without an option to escape or getting help, is also very common in PD [3].

Cognitive behaviour therapy (CBT) has been found to be an effective treatment for PD with and without agoraphobia, and most studies include CBT delivered with one or two weekly sessions [4–6]. According to Öst and Ollendick [7], concentrated treatment can be defined as interventions with more than one session/week during a fairly short time period. There is evidence to support that treatment can be delivered in more concentrated formats [5, 8]. For instance, a 2-day intervention [9], a five-day treatment [10] and a concentrated eight day format [11], which all showed promising results. In sum, emerging evidence suggests that the effectiveness of a standard CBT versus a brief, intensive and concentrated formats may be comparable [7, 12]. From a patient perspective, concentrated treatment formats may have some advantages and contribute to increased availability of treatment (i.e., travel distance to the clinic), faster recovery and lower dropout rates [13].

One concentrated and exposure-based format of CBT is the Bergen 4-day treatment (B4DT), which is delivered during four consecutive days. The treatment is designed to be an individual treatment delivered in a group setting, with a 1:1 ratio between therapists and patients. The focus of the treatment is to help patients recognize how they misinterpret benign bodily symptoms as signs of severe illness and that efforts to prevent the feared consequences through control of the symptoms may paradoxically maintain and intensify the problem. Exposure tasks are not designed as behavioural experiments to disconfirm catastrophic beliefs. Rather it is focused on emotional regulation, willingness and skills to let go of their efforts to control symptoms and the negative consequences they fear. This is done according to a procedure (“LEeaning in Technique” or “LET”) that focus on the willingness and how to “lean in” rather than hold back while doing exposure tasks.

The B4DT has been shown to be effective in treatment of obsessive–compulsive disorder [13–17], and a pilot study (*N* = 29) for PD has demonstrated promising results [18]. In this study 90% of patients achieved treatment response and 72% were considered to be in remission after three months [18]. These results have recently been replicated at a new site with 30 patients where 86% were considered in remission at three months follow-up [19]. A third and larger replication of B4DT treated 58 patients and found that 81% of patients were considered responders at three months follow-up, and 81% as in remission [20].

The aim of the current study is to investigate the feasibility, acceptability and effectiveness of the B4DT for PD when the treatment was delivered during the COVID-19 pandemic, where a significant portion of the treatment were delivered via videoconference.

## Method

### Participants and procedures

The study is a naturalistic investigation in a routine clinical setting, with no comparison group. The treatment was offered from April 2020 to May 2021 at the Clinic for 4-Day Treatment in Bergen, Norway, where the treatment format was developed. The study period was concurrent with the COVID-19-pandemic. All patients were referred to the Clinic for 4-Day Treatment and treatment was offered as a part of public health care. The study was approved by the regional committees for medical and health research ethics in Norway (REK-midt, ID 468517). All patients signed written consent for participation. A semi-structured interview using the panic disorder severity scale (PDSS) was performed before treatment, at post-treatment, and at three-months follow-up. Patients also completed questionnaires online before treatment (pre-treatment), one week after (post-treatment) and three-months after (follow-up).

The study sample included 50 patients who received treatment between April 2020 and May 2021. 41 patients (82%) were given a diagnosis of PD with agoraphobia, while 9 (18%) were given a diagnosis of PD without agoraphobia. Patients were not offered B4DT if they were suicidal, psychotic, actively abusing alcohol or narcotics, bipolar in manic phase, had a severe eating disorder or did not speak Norwegian.

### Assessment

#### Diagnostic interviews

The Mini International Neuropsychiatric Interview (MINI) was used to assess PD diagnosis and to screen for comorbid disorders [21]. The interview screens for axis-I DSM-IV disorders, and the Norwegian version has been shown to have sound psychometric properties [22].

Primary outcome measure. The PDSS is a seven-item interview to measure PD severity [23], and has been shown to have good psychometric properties [24]. Criteria presented by Furukawa [25] was used to define clinically significant change, where remission was defined as PDSS scores of five or less for patients without agoraphobia and scores at seven or below for patients with agoraphobia. Clinically significant change (response) was defined as a 40% or greater reduction [25]. The paper reports results from a study in routine clinical care, and no interrater assessment was performed. The one-week assessment was performed by an independent therapist in the same team, who did not participate in the treatment group, while the three-month assessment typically was carried out by the patient’s therapist from the group.

Secondary outcome measures. Secondary outcome measures consisted of six self-report scales. The Body Sensations Questionnaire (BSQ) was used to self-report fear of bodily symptoms, which are considered a core element of PD [26]. Mobility inventory for Agoraphobia (MI) was used as a measure of agoraphobic avoidance [27, 28]. The MI is divided in two scales, which respectively measures reduced mobility alone and together with others. Symptoms of depression were measured with the Patient Health questionnaire (PHQ-9; [29]). Symptoms of generalized anxiety were measured with the Generalized anxiety scale (GAD-7; [30]). Client Satisfaction Questionnaire (CSQ-8) was used to report treatment satisfaction, and has good psychometric properties [31]. Work and social impairment was measured by the Work and Social Adjustment Scale (WSAS; [32]). The score ranges from 0–40, where higher scores represent a larger impairment in work, daily chores, social activities, and ability to maintain personal relations. WSAS have been shown to have strong psychometric properties, in measuring impairment in work, social life and daily functioning [33].

### Treatment

The B4DT is a concentrated exposure-based treatment, which spans four days and is delivered to groups of 3–6 patients. The treatment is a combination of individually tailored exposure sessions and group sessions. The first day consists of patient education, with an emphasis on the case formulation for PD, factors that maintain the disorder and exposure technique to help break the pattern. The second and third days include therapist-assisted exposure to interoceptive symptoms relevant to the patient, and agoraphobic situations, with an emphasis on doing exposure with the “LEaning in Technique” (LET). Patients also shared experiences from the exposure sessions in the group, which was nuanced by repetition of the psychoeducation. The last day focuses on a summary of principles, facilitating lasting change and relapse-prevention. All patients make a three-week plan for self-exposure to consolidate the lessons learned as part of their everyday living. A more detailed description of the treatment structure and rationale is found elsewhere [18, 19].

As the treatment was delivered during the COVID-19 pandemic, adjustments were made to the treatment delivery to comply with national restrictions. This meant that a significant portion of the treatment was conducted via videoconference.

### Therapists

Five clinical psychologists experienced with CBT and the B4DT led the treatment groups. Five groups were led by one therapist, two other therapists led three groups each, while the last two led one group each. The group leaders had an average of 5.2 years (range 2–15) experience with treatment of anxiety disorders. Twenty-six therapists participated in treatment, and therapist competency and experience with the treatment format varied, as treatment was delivered as part of an implementation of B4DT in the region. All therapists had completed a two-day course in treatment of PD and were certified in the B4DT, which involves a clinical evaluation after participation on a minimum of two groups. The therapists were mainly psychologists, but also therapists from different health professions, such as nurses, psychiatrists, and psychology students.

### Statistical analyses

We used IBM SPSS Statistics (version 27) to perform statistical analyses. To investigate if the demographic variables were related to symptom severity prior to treatment, independent samples t-test and one-way ANOVAs was applied. A linear mixed model (LMM) design (with time as a fixed effect and intercept for each subject as a random effect) was used to investigate change in symptoms over time. With this model an increase in power is achieved as all available data can be used without imputation or listwise deletion [34, 35]. A diagonal covariance structure (heterogeneous variance and zero correlation between elements) was applied on the residuals, as this provided a good fit for the model. Parametric assumptions were assessed for each test and we found no indication that these assumptions were violated. Restricted Maximum Likelihood estimation with Satterthwaite approximation was chosen to estimate t- and p-values. Both primary and secondary outcomes were inserted into separate analyses using this same procedure. To reduce the probability of a type-I error when doing multiple independent analyses, a Bonferroni correction was performed, including the LMM analyses for primary (PDSS) and secondary (PHQ-9, GAD-7, BSQ, MI, WSAS). In order to investigate post-hoc differences, two dummy variables representing the two time intervals (pre-post and post-FU) was added as a covariate to the main analyses. Effect sizes were calculated as *Cohen’s d* = *(M*_*1*_* – M*_*2*_*)/SD*_*pooled*_ [36]. A pearson R correlation was used to investigate the correlation between the PDSS-scores and the self-report measures. The PDSS scores were compared to three previous studies on B4DT for PD by using independent samples t-tests.

## Results

### Feasibility

**Fig. 1:**
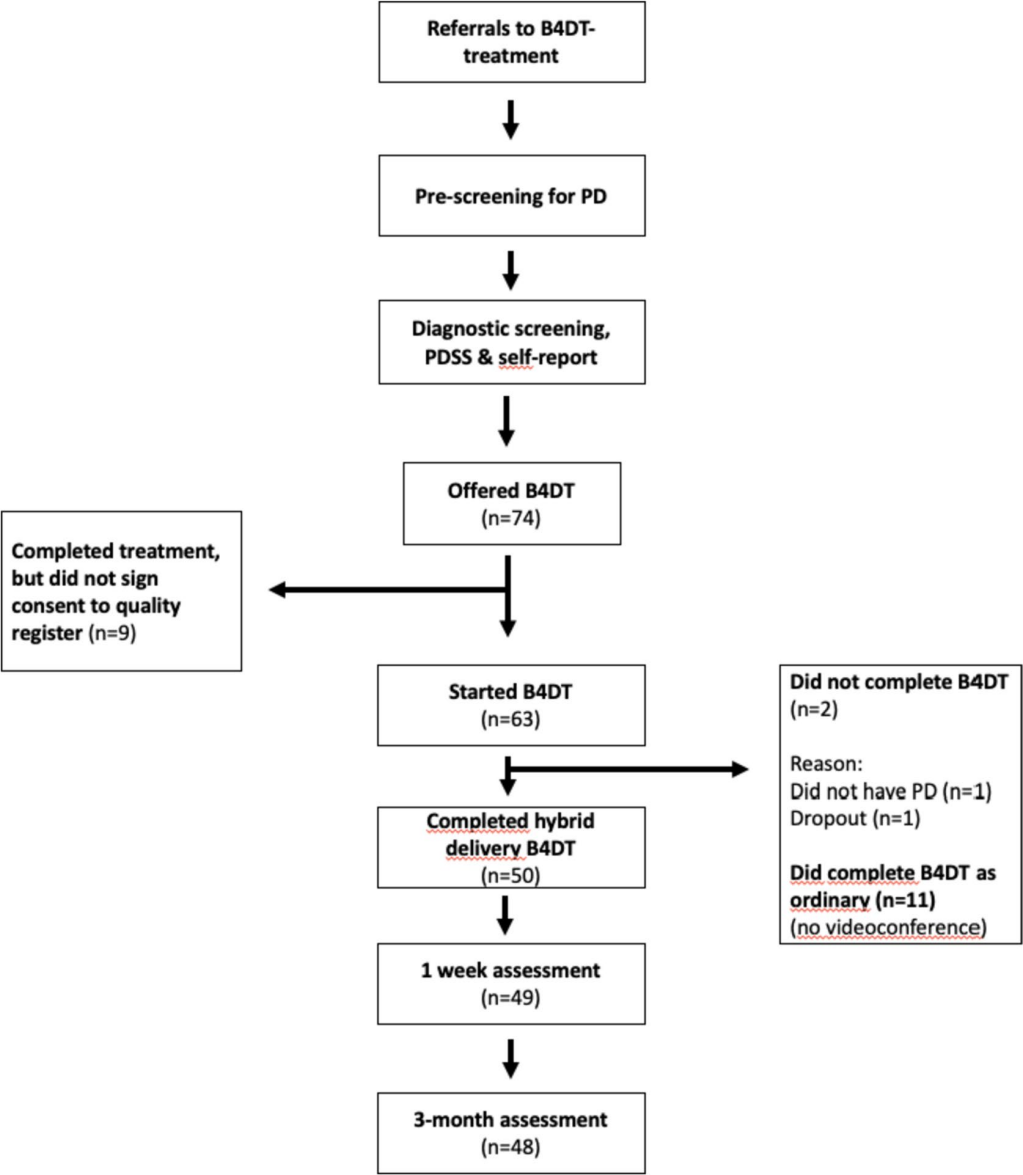
Flowchart B4DT, Bergen 4-day Treatment; PD, Panic disorder; PDSS, Panic Disorder Severity Scale

There were only one patient that dropped-out of the treatment (2%), and this patient was not included in the dataset. 50 patients were included in the analyses. The dataset had a low percentage of missing data, which for PDSS were only 2.% percent across the three assessment times (none at pre-treatment, 2% at post-treatment and 4% at follow-up). Self-report questionnaires were completed by all patients before treatment, 49 (98%) at 1 week and 43 (86%) at three months follow-up. See Fig. 1 for an overview of patient flow.

The treatment satisfaction of the treatment were high, as measured by an average score of 29.46 on the CSQ-8.

### Demographic information

A summary of patient characteristics and significance tests between demographic variables and PDSS at baseline is shown in Table 1. Twenty-four patients (48%) had at least one comorbid disorder, whereas 26 (52%) patients had no known comorbid disorder. Ten patients (20%) had major depressive disorder, seven (14%) social anxiety disorder, six (12%) generalized anxiety disorder, five (10%) obsessive–compulsive disorder, and three (6%) post-traumatic stress disorder. Baseline PDSS-scores for patients with comorbidity did not differ significantly from patients without a comorbid disorder, *t*(48) = -0.356, *p* = 0.36. Twenty-one (42%) patients reported using antidepressants, mainly SSRI and SNRIs, and these did not have a significantly higher PDSS at baseline compared to those who were not using antidepressants, *t*(48) = 3.1, *p* = 0.42. No changes were done to SSRI during active treatment period, and patients were on a stable dose at least four weeks before the time of treatment. Five patients (10%) used benzodiazepines sporadically and were instructed to not use these in the treatment period. No patients were in the acute phase of PD, and the duration of the disorder ranged from 6 months to 40 years.

**Table 1:** Characteristics and relation with PDSS at baseline

Variable	M (SD)	*F* (df)	*p*
Age (years)	32.7 (9.4)	.57 (27,22)	.92
Duration of the disorder (months)	90.9 (100.0)	1.13 (18,30)	.37
	N (%)	*t* (df)	*p*
Gender: females	34 (68)	.58 (48)	.28
Have received previous treatment	32 (64)	.52 (48)	.30
Care for children	19 (38)	.36 (48)	.36
	N (%)	*F* (df)	*p*
Marital status		.85 (2,47)	.43
Single	14 (28)		
Married or cohabiting	35 (70)		
Partner (not cohabit)	1 (2)		
Educational status		1..91 (2,47)	.37
Junior high school	7 (14)		
High school	25 (50)		
College	18 (36)		
Work status		2.40 (3,47)	.08
Working / studying full time	30 (60)		
Working / studying part time	4 (8)		
Sick leave	14 (28)		
Welfare / pension	2 (4)		
Comorbidity	24 (48)	.13 (1,48)	.72
No comorbidity	26 (52)		
Comorbid anxiety	16 (32)		
Comorbid depression	11 (22)		
Psychotropic medication	24 (48)	5.30 (1,48)	.03
No medication	26 (52)		
SSRI / SNRI	21 (42)		
Benzodiazepines	5 (10)		

#### Primary outcome measure

**Fig. 2:**
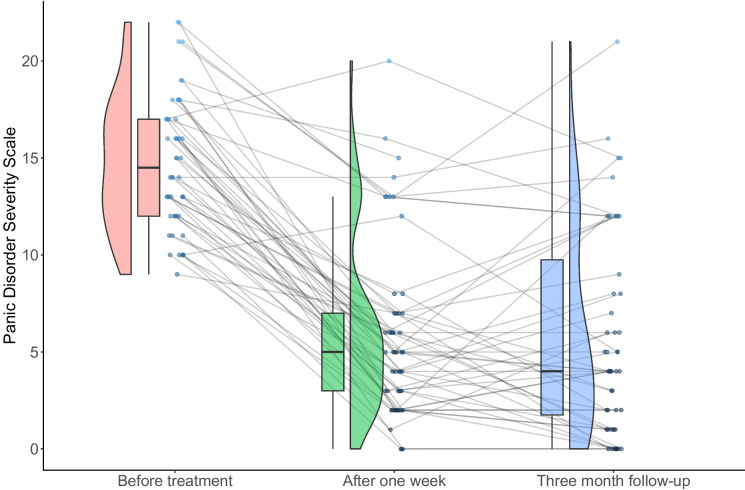
PDSS trajectories across time points *Note.* Raincloud plot visualizing PDSS scores for all patients across the three time points. The figure shows the distribution of scores, and the box plot visualizes the standard deviation, median, interquartile range and 95% confidence interval

Mean scores of the dependent measures and effect sizes across all time points are found in Table 2. The PDSS showed a significant effect of time (F(2,50) = 140.99, *p* < 0.001), indicating significant change over time/treatment. This significant reduction in PDSS-scores was found from pre- to post-treatment, (F(2,50) = 206.56,* p* < 0.001, Cohen’s d(*d*) = 2.18, but not between post and follow-up, F(2,50) = 0.03, *p* = 0.863, d(*d*) = 0.041. At one week follow-up, a total of 74% was in remission, while 82% had a clinically significant change. Three months after treatment, a total of 62% of patients were in remission, while 70% was considered to have a clinically significant change. The Bonferroni analyses showed that the primary and secondary outcome measures were still below < 0.001 significance level after accounting for multiple comparisons. PDSS-scores for all patients across the three time-points are illustrated in Fig. 2.

**Table 2:** Results and effect size on primary and secondary outcome measures

*Variable*	*Pre*	*Post*	*F-up*	*ES Pre—post*	*ES Pre—f-up*
PDSS	14.78 (3.37)	6.08 (4.52)	5.88 (5.28)	2.18	2.01
BSQ	3.03 (0.61)	2.33 (0.72)	2.14 (0.83)	1.04	1.22
MI, Comp	1.93 (0.76)	1.57 (0.71)	1.52 (0.69)	0.49	0.56
MI, Alone	2.59 (1.00)	1.93 (0.90)	1.92 (0.94)	0.69	0.69
GAD-7	10.58 (4.70)	6.24 (4.50)	6.58 (5.23)	0.94	0.80
PHQ-9	10.62 (4.80)	7.88 (5.40)	7.49 (4.81)	0.54	0.65
WSAS	16.66 (8.10)	12.16 (9.16)	11.33 (9.85)	0.52	0.59
CSQ-8		29.46 (2.88)			

*PDDS*, Panic Disorder Severity Scale, *BSQ* Body Symptom Questionnaire, *MI* Mobility Inventory for Agoraphobia, scores with companion and alone, *GAD-7* Generalized Anxiety Disorder-7, *PHQ-9* Patient Health Questionnaire-9, *WSAS* Work and social adjustment scale, *CSQ-8* Client satisfaction questionnaire, *ES* Cohen’s d

#### Secondary outcome measures

There was a significant decrease in secondary symptoms across all measures after treatment, and the correlations between the reduction in PDSS-scores and self-report measures are found in Table 3. Secondary measures of PHQ-9 and GAD-7 showed an effect of time/treatment (PHQ-9, F(2,50) = 20.09, *p* < 0.001; GAD-7, F(2,50) = 27.39, *p* < 0.001). The reduction in PHQ-9 was significant only from pre- to post-treatment (*F*(2,50) = 17.71, *p* < 0.001, Cohen’s d (*d*) = 0.54) and not from post-treatment to FU (*F*(2,50) = 0.30, *p* = 0.588, d(*d*) = 0.08). Post-hoc test for GAD-7 showed a significant decrease from pre- to post-treatment (*F*(2,50) = 26.47, *p* < 0.001, d(*d*) = 0.94) but not between post-treatment and FU (*F*(2,50) = 0.43.11, *p* = 0.516,* d*(*d*) = 0.07). BSQ (F(2,50) = 40.36, *p* < 0.001), MI avoidance-alone (F(2,50) = 26.62, *p* < 0.001) and MI avoidance-accompanied (F(2,50) = 13.64, *p* < 0.001) also showed a significant effect of time/treatment. Post-hoc tests of BSQ, MI avoidance-alone and MI Avoidance-accompanied also showed significant reductions from pre- to post-treatment (BSQ, *F*(2,50) = 47.22, *p* < 0.001, d(*d*) = 1.05; MI avoidance-alone, *F*(2,50) = 39.36*, p* < 0.001, d(*d*) = 0.69; MI avoidance-companion, *F*(2,50) = 16.32, *p* < 0.001, d(*d*) = 0.49), but not between post-treatment and FU (BSQ, *F*(2,50) = 2.54, *p* = 0.117, *d*(*d*) = 0.24; MI avoidance-alone, *F*(2,50) = 2.27,* p* = 0.14, d(*d*) = 0.01; MI avoidance-accompanied, *F*(2,50) = 3.12, *p* = 0.085, *d*(*d*) = 0.07). On average, the patients reported increased daily function after treatment, as we found an effect of time/treatment on WSAS (F(2,50) = 15.81, *p* < 0.001). The significant increase in functioning (i.e. decrease in WSAS) was found from pre- to post-treatment (F(2,50) = 23.84, *p* < 0.001, d(*d*) = 0.52), but not from post-treatment to FU (F(2,50) = 0.73, *p* = 0.399, d(*d*) = 0.09) (Fig. 2).

**Table 3:** Total score correlations with 95% confidence intervals

Measure	PDSS	95% confidence interval
PHQ-9	.276	-.030-.535
GAD-7	.334*	.033-.579
BSQ	.438**	.154-.654
MI-companion	.421**	.130-.645
MI-alone	.326*	.025-.573

*PDDS* Panic Disorder Severity Scale, *BSQ* Body Symptom Questionnaire, *MI* Mobility Inventory for Agoraphobia, *GAD-7* Generalized Anxiety Disorder-7, *PHQ-9* Patient Health Questionnaire-9

^*^p < .05, **p < .01

### Comparison with previous studies

PDSS scores from pre, post and follow-up were compared to the three previous B4DT studies on PD (see Table 4). We found that the pre-treatment PDSS scores was not significantly different from the other B4DT studies, except Iversen and colleagues [19], *t*(77) = 6.37, *p* < 0.001. At one week post, the PDSS scores of the current study did not differ from the three previous studies on PD. At three-months follow-up, PDSS scores of the current study did not differ from the pilot study [18], *t*(77) = 0.954, *p* = 0.343, but it was higher than Eide and colleagues [20], *t*(106) = 3.64, *p* < 0.001, and Iversen and colleagues [19], *t*(78) = 2.988, *p* = 0.004.

**Table 4:** Comparison of PDSS-scores to previous B4DT studies on PD

	Hansen et al. [18] *N* = 29	Iversen et al. [19] *N* = 30	Eide et al. [20] *N* = 58	Current study *N* = 50
Pre	15.79 (3.97)	19.83 (3.44)	16.10 (3.90)	14.78 (3.37)
Post	5.34 (4.22)	4.37 (3.72)	4.72 (2.77)	6.08 (4.52)
ES Pre-post	2.55	4.32	3.36	2.18
FU	4.82 (3.65)	2.62 (3.57)	2.81 (3.38)	5.88 (5.29)
ES Pre-f-up	2.88	4.91	3.64	2.01

*PDDS* Panic Disorder Severity Scale, *ES* Cohen´s d compared to pre

## Discussion

In this study, we examined the feasibility, acceptability and effectiveness of the B4DT for PD adapted to COVID-19 restrictions, as a substantial proportion of the treatment were delivered by videoconferencing. The results indicates that using videoconference formats in the delivery of concentrated exposure therapy can be a promising adaptation, especially when face-to-face treatment is difficult due to COVID-19 restrictions, geographical distance, or challenges with in-person meetings. We found a substantial effect of treatment on PD severity (as measured by Panic Disorder Severity Scale) and secondary measures including depression, anxiety, and work and social adjustment.

The results are comparable to the results from the B4DT pilot study [18]. However, the PDSS scores from the present study were somewhat higher at follow-up than the results from the two B4DT replication studies [19, 20]. These studies were conducted with highly experienced B4DT-therapists and before the COVID-19 pandemic. In contrast, the current study was conducted during the pandemic and included 26 different therapists. We also found a substantial effect on BSQ and MI, although the effect was somewhat smaller than for PDSS. This is consistent with previous studies that have reported lower effect sizes for MI and BSQ compared to PDSS [37]. There should also be noted that the PDSS is administered by clinicians, while the PDSS was self-report, which may have influenced the reporting. Since MI measures mobility in different settings, the fact that the treatment was conducted under COVID-19 restrictions, which limited the ability to visit public places alone or with others, may have affected the results. BSQ which assesses fear for bodily sensations, may also have been affected by the COVID-19 period, where there may have been more anxiety about bodily sensations, since the listed symptoms might also be symptoms of COVID-19. BSQ, which assesses fear of bodily sensations, may also have been influenced by the COVID-19 period. During this time, there may have been increased anxiety about bodily sensations, as some of the listed symptoms could also be symptoms of COVID-19. Further studies should therefore be done in different settings to explore the possible effect of PDSS of MI and BSQ.

The difference in results may be related to the pandemic and some reports indicate that there has been a significant increase in symptoms of anxiety and depression during the pandemic [38, 39]. Still, we did not find increased baseline symptoms in our sample when comparing symptoms of PD prior to treatment to the previous studies. There is also a chance that the pandemic might have affected the consolidation period following the treatment. In contrast to studies pre-COVID we did find a reduction in remission rate from post-treatment (72.1%) to follow-up (62.3%), and long-time follow-up of this sample is thus warranted. Some patients with PD may also be more vulnerable for relapse during the COVID-pandemic, since the virus affects the respiratory system which also is where the fear originates for many PD-patients [40].

Overall the results are consistent with previous literature showing that traditional CBT for anxiety disorder could be equally effective when delivered in a combination of videoconferencing and face-to-face [25, 26]. Our findings show that concentrated CBT such as the B4DT can be adapted to include videoconferencing, although the results are somewhat lower than in previous studies on B4DT. There is still need for more studies to establish the effectiveness of B4DT, but it seems to be a promising CBT-format in the treatment of severe anxiety disorders such as panic disorder [18, 19], and for obsessive compulsive disorder [13–17, 41]. The structure of the B4DT may also increase adherence and facilitate therapist competency, as it involves multiple check in with colleagues, observation by colleagues, and almost continuous monitoring of the entire treatment by both patients and therapists.

As the present study is a naturalistic report from ordinary mental health care, and as is common in settings like this, it has its limitations. There was no comparison group and thus we could not compare our results directly to treatment as usual or other treatments for PD. The study also has a low number of participants, that could enhance the effect size-estimates, thus overestimating the effectiveness of the treatment. We note that even if the results are highly encouraging, there is not yet an RCTs comparing B4DT for PD with other active treatment formats and thus not a basis to draw conclusions regarding the relative effectiveness of different formats compared to each other. The study did not include measures of treatment fidelity and competence, adherence, or compliance, and therefore could not investigate if variation in these variables was related to outcome. The high number of therapists caused by the concurrent implementation of B4DT, could also affect the therapist competency and accumulated experience of each therapist. On the other side, as there are 3–6 therapists in each group working together, this may increase adherence to the treatment manual. A strength of the treatment is the delivery in an outpatient clinic during the COVID-pandemic, which may strengthen the ecological validity of the findings. In relation to the assessment aspect, it is important to highlight that the study lacks inter-rater assessment for the clinically administered instruments. Additionally, it is worth noting that the clinicians involved in the treatment were responsible for conducting the three-month PDSS interviews, which raises the possibility of their influence on the patients' responses. These assessment procedures impose limitations on the conclusions that can be drawn from the study. In the future, long-term follow-up and more strictly controlled studies, preferably with active controls are warranted.

## Conclusion

The current study supports B4DT as an effective treatment for PD, even when delivered in a combination of videoconference and face-to-face meetings. We conclude that it seem both feasible and useful to deliver concentrated exposure therapy by hybrid formats.

## Data Availability

Data are anonymously available from the corresponding author on reasonable request.
